# Tumor-associated macrophages: an accomplice in solid tumor progression

**DOI:** 10.1186/s12929-019-0568-z

**Published:** 2019-10-20

**Authors:** Yibing Chen, Yucen Song, Wei Du, Longlong Gong, Haocai Chang, Zhengzhi Zou

**Affiliations:** 1 Genetic and Prenatal Diagnosis Center, Department of Gynecology and Obstetrics, First Affiliated Hospital, Zhengzhou University, 1 Jianshe Road East, Zhengzhou, 450052 Henan China; 2 Department of Neurosurgery, First Affiliated Hospital, Zhengzhou University, Zhengzhou, 450052 China; 3 MOE Key Laboratory of Laser Life Science and Institute of Laser Life Science, College of Biophotonics, South China Normal University, Guangzhou, 510631 Guangdong China

**Keywords:** Tumor-associated macrophages, Solid tumor, Tumor growth, Chemotherapy and radiotherapy resistance, Angiogenesis, Migration, Invasion, Metastasis, Immunosuppression, Therapeutic target

## Abstract

In many solid tumor types, tumor-associated macrophages (TAMs) are important components of the tumor microenvironment (TME). Moreover, TAMs infiltration is strongly associated with poor survival in solid tumor patients. In this review, we describe the origins of TAMs and their polarization state dictated by the TME. We also specifically focus on the role of TAMs in promoting tumor growth, enhancing cancer cells resistance to chemotherapy and radiotherapy, promoting tumor angiogenesis, inducing tumor migration and invasion and metastasis, activating immunosuppression. In addition, we discuss TAMs can be used as therapeutic targets of solid tumor in clinics. The therapeutic strategies include clearing macrophages and inhibiting the activation of TAMs, promoting macrophage phagocytic activity, limiting monocyte recruitment and other targeted TAMs therapies.

## Background

**Fig. 1:**
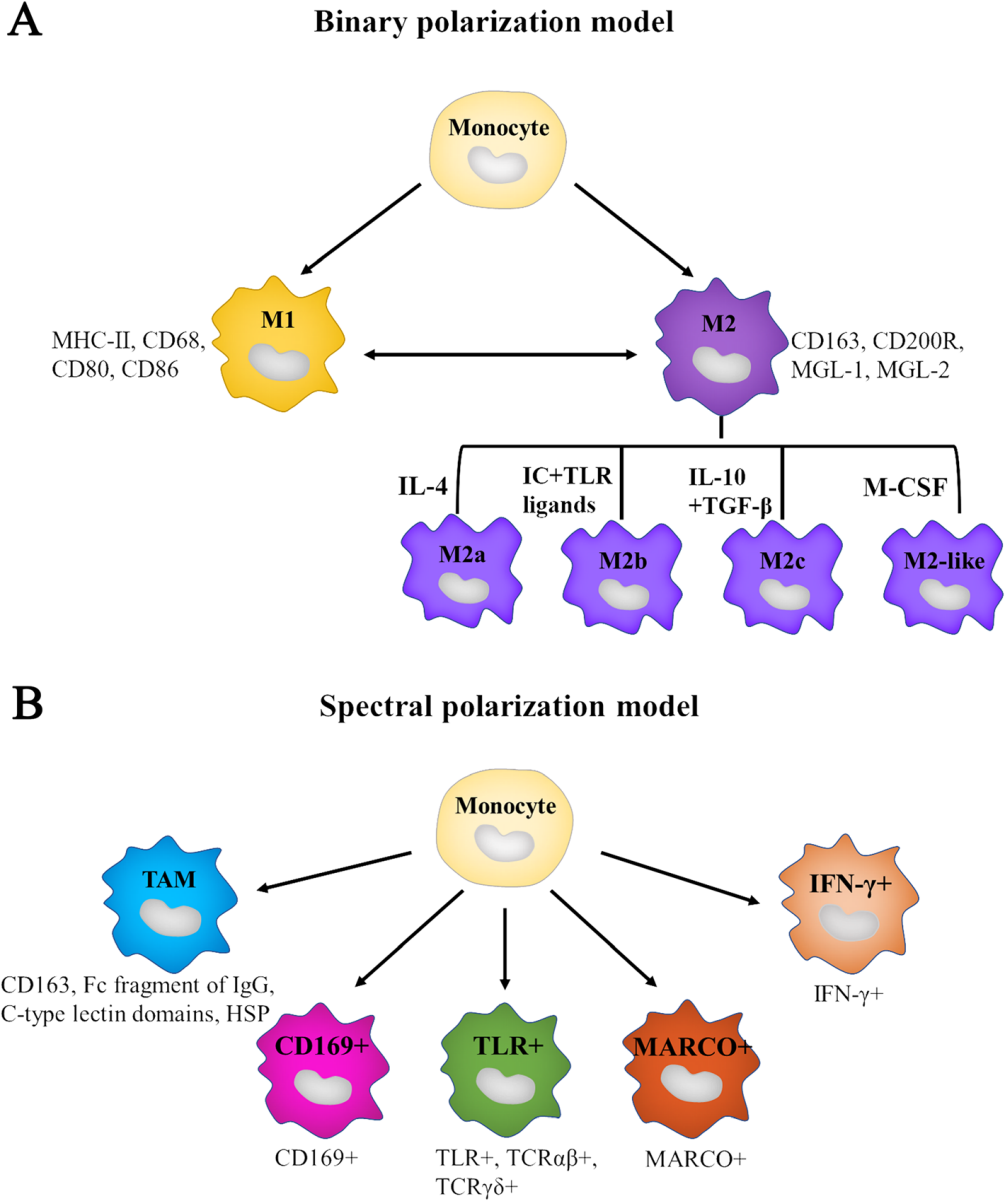
The polarization model of macrophage from monocyte differentiation. **a**. In conventional binary model, macrophages polarize into two subtypes, M1 macrophages with marker such as MHC-II, CD68, CD80, CD86 and M2 subtypes with marker such as CD163, CD200R, MGL-1, MGL-2. In response to different factors stimulation, M2 polarization is represented by four different phenotypes, including M2a (IL-4), M2b (immune complexes (IC) and TLR ligands), M2c (IL-10 and TGF-β) and M2-like (growth factors and cytokines in TME like M-CSF). **b.** In spectral polarization model, monocytes are differentiated into different subtypes of macrophages with different marker. The surface of TAMs marked with CD163, Fc fragment of IgG, C-type lectin domains and HSP. Other subtypes of macrophages have different marker such as CD169, TLR, MARCO, IFN-γ

Solid tumor development and progression are complex processes, which are not only induced by accumulated genetic mutants in cancer cells, but also regulated by the surrounding microenvironment. Much noticeable evidence shows that the tumor microenvironment (TME) engage in cancer initiation and promotion of tumor growth [1]. TME comprise innate and adaptive immune cells such as T cells, dendritic cells and macrophages in solid tumor. Macrophages are roughly classified into three populations, including tumor-associated macrophages (TAMs) derived from mononuclear cells, tissue-resident macrophages and myeloid derived suppressor cells (MDSC). TAMs are the most abundant population of tumor-infiltrating immune cells in TME [2]. Macrophages are extremely plastic cells. They have two polarization states: classically activated M1 and alternatively activated M2 subtypes (Fig. 1a) [3]. Th1 cytokines such as interleukin-12 (IL-12) and IL-18 or activated Toll-like receptors (TLRs) promote macrophages to M1 polarization. M1 macrophages are involved in Th1 responses to pathogens [4]. M1 macrophages play critical roles in innate host defense and killing tumor cell by producing reactive oxygen/nitrogen species (ROS/RNS) and pro-inflammatory cytokines such as IL-1β, IL-6, tumor necrosis factor α (TNF-α). Therefore, they are considered as antitumor or “good” macrophages [4]. On the other hand, macrophages are induced polarization into the M2 by Th2 cytokines such as IL-4, IL-10 and IL-13. M2 macrophages are crucial for Th2 immune response including humoral immunity, wound healing and tissue remodeling. Moreover, M2 macrophages produce anti-inflammatory cytokines such as IL-10, IL-13 and TGF-β to promote tumor development. Therefore, they are considered as pro-tumor or “bad” macrophages. M2 macrophages are represented by four phenotypes, including M2a, M2b, M2c and M2-like (Fig. 1a). M2a macrophages are activated by IL-4 with Th2 immune response. M2b macrophages exert immunoregulatory roles under the activation of immune complexes (IC) and TLR ligands. M2c macrophages are polarized by IL-10, and play roles in immunoregulation and tissue modelling. M2-like macrophages activated by growth factors and cytokines in TME are considered to be M2d subtype with immunosuppressive role and protumor property [5]. Macrophage colony-stimulating factor (M-CSF)-induced macrophages play important roles in participating in homeostatic and pathological process [4]. Apart from the classical binary polarization model, a novel spectral polarization model is also put forward. The spectral polarization model points out monocyte differentiated into different subtypes of macrophages with different markers such as CD169, TLR, MARCO, interferon-γ (IFN-γ) (Fig. 1b). The different subtypes of macrophages in spectral polarization model exert important roles in various human pathologies.

**Fig. 2:**
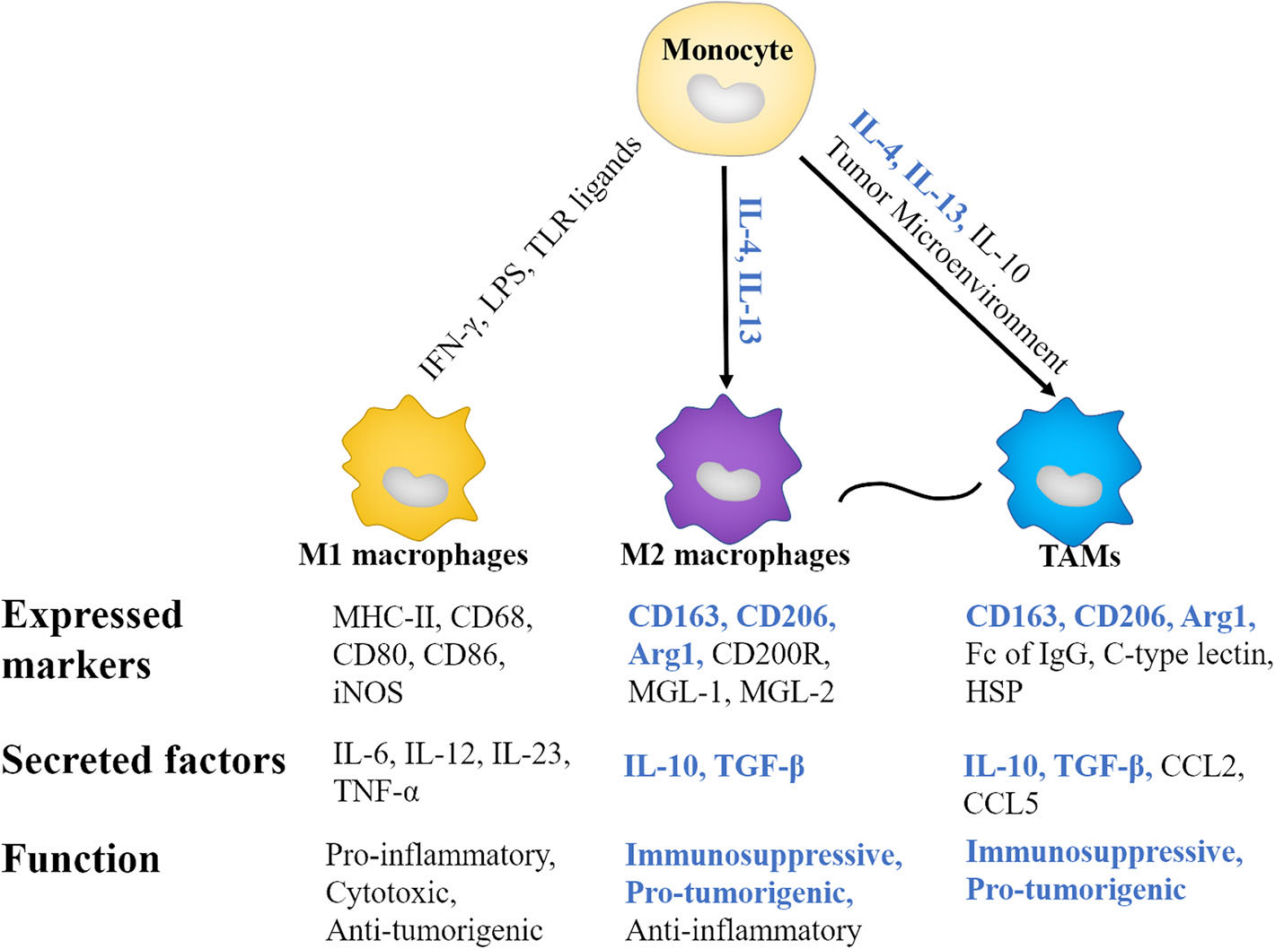
Macrophage differentiation and their characteristics. M1 macrophages are activated by IFN-γ, LPS or TLR ligands, expressing MHC-II, CD68, CD80, CD86, and secreting IL-6, IL-12, IL-23, and exerting pro-inflammatory, cytotoxic and tumoricidal roles. On the contrary, M2 macrophages and TAMs exert immunosuppressive and pro-tumorigenic roles. In general, TAMs are thought to more closely resembleM2 macrophages. Both TAMs and M2 macrophages are activated by helper T cell 2 cytokines IL-4 and IL-13, expressing CD163, CD206, Arg1, and secreting IL-10 and TGFβ. However, TAMs show some characteristics different from M2 macrophages. For example, TAMs express Fc of IgG, C-type lectin, HSP, and secret CCL2 and CCL5.M2 macrophages express high levels of MGL1 and MGL2, members of the macrophage galactose type C-lectin family

**Fig. 3:**
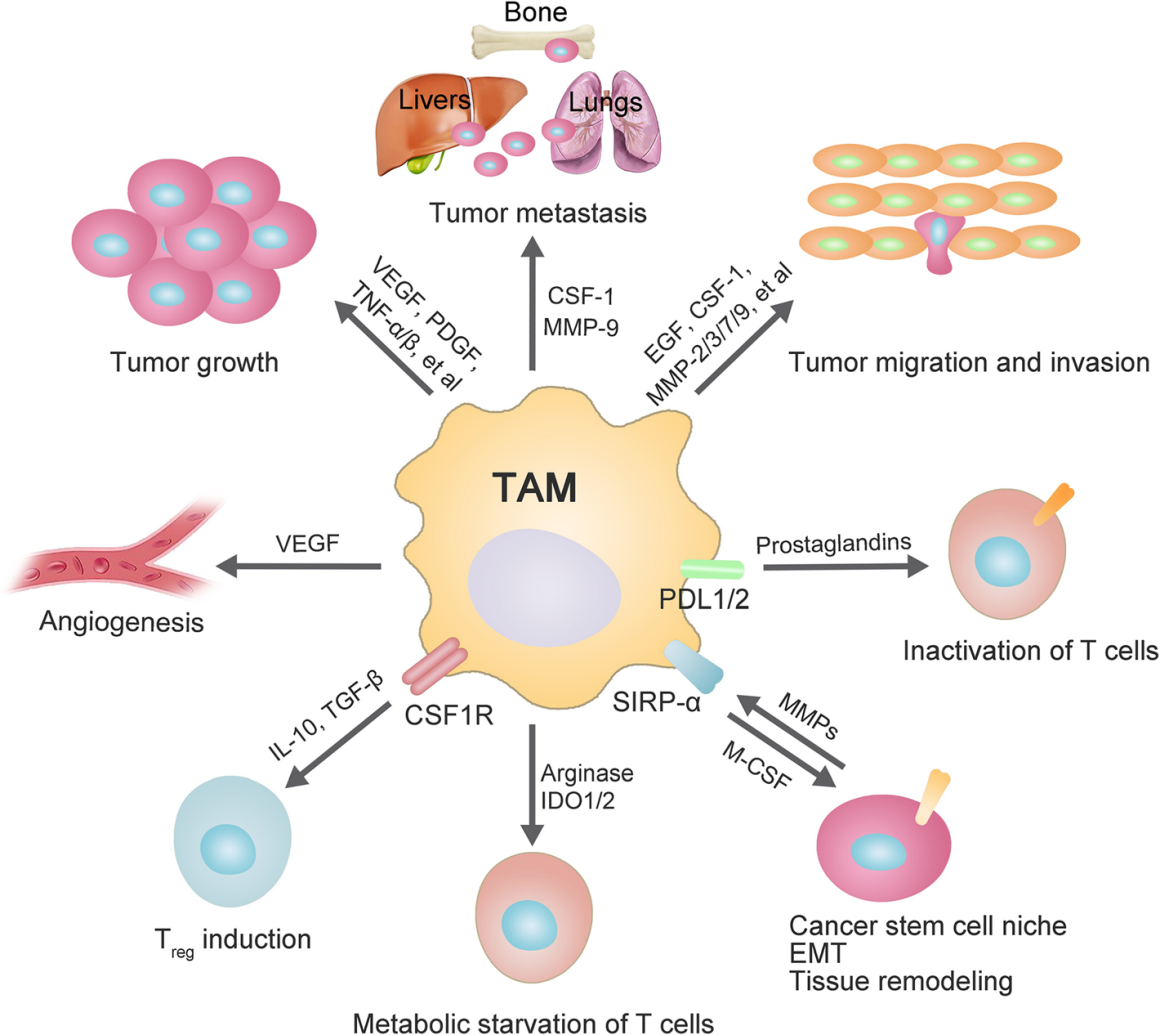
Main roles of tumor-associated macrophages in tumorigenesis. Special markers and different factors derived from TAMs trigger multiple processes of tumor initiation and development. Schematic represents the impact of the tumor-associated macrophages (TAMs) in promoting tumor growth, angiogenesis, Treg cells induction, metabolic starvation of T cells, cancer stem cells induction, T cells inactivation, epithelial-mesenchymal transition (EMT), invasion, migration and metastasis. TAMs promote tumorigenesis by secreting some factors and expressing some proteins. For instance, epidermal growth factor, CSF-1 and MMPs secreted by TAMs promote tumor migration and invasion. TAMs promote angiogenesis and tumor growth by secreting VEGF and PDGF

Macrophages are the important part of the immune system and found in almost all tissues. TAMs are consisted of two major cell subtype populations classified as either M1 or M2 macrophages [2]. Generally, TAMs are thought to closely resemble M2 macrophages with Th2 immune response (Fig. 2). Macrophages are phagocytic in nature and play indispensable roles in homeostasis and defense [6]. However, in many solid tumors, it has been found that high densities of cells with macrophage-associated markers are related to a poor clinical outcome [7]. As shown in Fig. 3, TAMs play major roles in tumor initiation, growth, development and metastasis by secreting a wide variety of cytokines, growth factors, inflammatory substrates and proteolytic enzymes (Table 1). In this review, we described the current knowledge about major roles of TAMs in cancer progression, and summarized current solid tumor therapeutic strategies by targeting TAMs. Our review would help to shed light on the ways to target TAMs for therapeutic interventions, as well as potential of TAMs as prognostic biomarkers for various solid tumors.

**Table 1:** The cytokines, chemokines and enzymes derived from TAMs and associated signaling pathways

	Signaling pathway	Process	Solid tumor	Derivation	Ref.
Cytokines					
IL-1	IL-1β/15-PGDH	Tumor growth	Pancreatic cancer	M1, TAM	11
	PGDF	Neovascularization	Colon cancer		37
IL-6	IL-6/STAT3	Chemotherapy resistance	Colorectal cancer and pancreatic ductal adenocarcinoma	M1, TAM	13
		Neovascularization	Colon cancer		37
IL-10		Suppress T cells		M2, TAM	78
TNF-α	NF-κB/MITF	Chemotherapy resistance	Melanoma	M1, TAM	34
TGF-β	Smad/Snail signaling pathway	EMT	Colorectal cancer	M2a, TAM	65
	TGF-β/SOX9	Cell invasion	Non-small cell lung cancer		52
VEGF	HIF1α	Neoangiogenesis	Skin cancer mouse model and Merkel cell carcinoma	TAM	35,41,42
Chemokines					
CXCL8	CXCL8/HOXB13/ERα	Invasion and metastasis	Endometrial cancer	TAM	9
TIE2	ANG2/TIE2	angiogenesis	Mammary tumor and pancreatic insulinoma models	TAM	45
CCL18	CCL18/ PITPNM3	Migration	Breast cancer	M2, TAM	54
	GM-CSF/CCL18 feedback loop	EMT and metastasis	Breast cancer		67
CCL20		Recruit CCR6 + nTreg	Colorectal cancer	M1, M2b, TAM	77
CCL22		Recruit CCR4 + nTreg	Ovarian carcinoma	M2, TAM	76
Enzymes					
Cathepsin	Cathepsin B/Nlrp3	Tumor growth, invasion and metastasis	Breast cancer	TAM	33,50
MMPs	MMP-9	Angiogenesis and tumor growth	Melanoma	TAM	12
	MMP-9	Tumor migration and invasion	Triple-negative breast cancer mouse model and lung cancer	TAM	55,56

## Role of TAMs in tumor growth

The importance of the TAMs in promoting tumor initiation and development has been increasingly recognized. TAMs not only directly provide structural support for cancer development, but also participate in tumor initiation by secreting signal molecules and extracellular vesicles (EVs) [8]. Those signal molecules secreted by macrophages contain growth factors, cytokines and chemokines, such as transforming growth factor β (TGF-β), vascular endothelial growth factor (VEGF), platelet derived growth factor (PDGF), M-CSF, IL-10 and chemokine C-X-C motif ligand (CXCL). For example, TAMs in hepatocellular carcinoma contribute to tumor development by inducing hepatocyte growth factor (HGF) expression [9]. Additionally, TAM-secreted CXCL8 promotes tumor progress by decreasing ERα expression via HOXB13 in endometrial cancer [10]. Lindsten et al. also showed that macrophages can decrease ERα and progesterone receptor (PR) expression in breast tumor cells, whereas increase uPAR and Ki67 expression. Moreover, they suggested that TAMs are associated with poor prognosis in breast cancer patients [11]. In pancreatic ductal adenocarcinoma, TAMs prevent 15-hydroxyprostaglandin dehydrogenase (15-PGDH) expression by secreting IL-1β and are associated with poor prognosis of patients [12]. In numerous cancers, such as liver cancer, breast cancer, renal cell carcinoma, Hodgkin lymphoma and ovarian cancer, elevated production of M-CSF by TAMs has been found to be associated with cancer development. By contrast, low expression of M-CSF is found in normal ovarian and breast tissues [13].

Tumor stem cells (TSC), the specific cancer cell subpopulations with stem cell-like properties, exert the potential to initiate tumorigenesis by undergoing continuous self-renewal and differentiation [14]. There are growing evidences to support that TAMs directly communicate with TSC to promote their survival and subsequent tumorigenesis. TAMs secret growth factors to support TSC survival, self-renewal and maintenance. In turn, TSC provide pivotal tumor-promoting signals to activate TAMs that further promote tumorigenesis [15]. For example, Jinushi et al. reported that TAMs interact with TSC and increase their tumorigenic potential by inducing milk-fat globule-epidermal growth factor-VIII (MFG-E8) and IL-6 to activate STAT3 and sonic hedgehog signal pathways. Conversely, TSC promote macrophages to produce MFG-E8 and IL-6 [16]. In hepatocellular carcinoma, TAMs promote stem cell-like properties of cancer cell via TGF-β [17]. In glioblastoma, periostin secreted by TSC recruits monocyte-derived macrophages from peripheral blood to induce M2 TAMs and promote malignant growth [18]. In addition, Raghavan et al found that ovarian TSC and TAMs reciprocally interact through the WNT pathway in 3-dimension engineered microenvironments [19].TSC educate monocytes towards pro-tumoral TAMs, and the TSC-educated TAMs reciprocally enhance the stem-like properties of ovarian cancer cells and malignant phenotypes [19].

## TAMs enhance cancer cells resistance against chemotherapy and radiotherapy

Chemotherapy and radiotherapy are two main treatments for solid cancer. However, cancer cells resistance to these two treatments is a common phenomenon, especially in progressive solid tumors [20–24]. Growing evidence has shown that TAMs are involved in cancer chemotherapy and radiotherapy resistance. Depletion or inhibition of TAMs can attenuate chemotherapy and radiotherapy resistance in vivo and in vitro. For instance, Paulus et al. reported inhibition of macrophages by using a colony stimulating factor 1 (CSF-1) neutralizing antibody increases chemosensitivity in human breast cancer xenografts [25]. In an orthotopic prostate cancer mode, targeting TAMs using CSF-1 receptor inhibitor effectively ameliorate tumor development and androgen deprivation therapy resistance [26]. Additionally, macrophage depletion with either CSF-1 antibody or CSF-1 receptor kinase inhibitor (i.e. PLX3397) significantly reduces tumor regrowth followed by radiotherapy in mice bearing mammary tumor [27]. In a prostate cancer model, Xu et al. investigated the effects of TAMs in tumor-bearing animals after irradiation. They found CSF1R inhibitor combined with irradiation suppresses tumor growth more effectively relative to irradiation alone. Their results imply that macrophages can limit the efficacy of radiotherapy [28].

TAM-secreted cytokines induce anti-apoptotic programs in cancer cells. IL-6, a pleiotropic cytokine, plays an important role in the regulation of immune system. Moreover, TAMs-derived IL-6 mediates resistance of solid tumor to many chemotherapy drugs [29]. For example, inhibition of Hedgehog pathway significantly induces IL-6 expression of macrophages. The upregulation of IL-6 expression mediates tolerance to chemotherapy drugs in breast cancer [30]. In addition, in colorectal cancer, TAMs-derived IL-6 activates the STAT3 pathway, and activated STAT3 transcriptionally blocks the tumor suppressor miR-204-5p expression. The attenuation of miR-204-5p promotes chemotherapy resistance by increasing anti-apoptotic protein RAB22A and bcl2 expression in cancer cells [31, 32]. In pancreatic ductal adenocarcinoma (PDAC), STAT3 activation in TAMs is necessary for macrophage-dependent gemcitabine resistance. Furthermore, induction of IL-6 and GP130 transcription mediated by STAT3 may be involved in the chemotherapy resistance [33]. Masahisa et al. clarified MFG-E8 in TAMs regulating TSC activities. Moreover, MFG-E8 amplifies cancer cells resistance to cisplatin in cooperation with IL-6 by activating STAT3 and Sonic Hedgehog signal pathways [16]. Other cytokines such as IL-10 and IL-34 are reported to mediate resistance to chemotherapy in many solid tumors. Ruffell et al. found IL-10 expressed by TAMs is the critical mediator in tumor resistance to paclitaxel and carboplatin in transgenic mouse luminal B-type mammary carcinoma model [34]. In addition, chemotherapy-induced IL-34 enhances TAM-mediated chemoresistance in lung cancer [35]. In mammary (breast) tumors, TAM-secreted IL-4 was reported to limit efficacy of radiotherapy [27]. Xu et al. found that TAMs suppress the efficacy of radiotherapy by secreting CSF1 to enhance CSF1R signaling in prostate cancer [28].

Other survival factors secreted by TAMs have also been reported to induce chemoresistance in cancer [36]. TAMs in hepatocellular carcinoma contribute to chemoresistance by inducing HGF [37]. TAMs have been reported to directly promote the survival of breast cancer cells by secreting cathepsin in vitro [38]. Surprisingly, cathepsin proteases in TAMs, specifically cathepsin B and S, contributes the production of soluble chemoprotective factors. Therefore, inhibition of cathepsin enhances the response of mammary carcinoma to paclitaxel in vivo [38]. MDSC-derived TAM release cathepsin B to activate the Nlrp3 inflammasome to promote tumor growth [39]. This raises a possible underlying mechanism that Nlrp3 inflammasome activated by cathepsin B contributes chemoresistance. The RAF/MEK/ERK mitogen activated protein kinase (MAPK) signaling pathway is hyperactivated in melanomas. This MAPK pathway-targeted therapy by selectively inhibiting the RAF kinase has been utilized successfully in the clinic. However, the resistance to MAPK pathway-targeted therapy activated by immune-microenvironment limits the efficacy of tumor therapy. Recently, Michael et al. identified TAMs-derived TNFα promotes melanoma resistance to MAPK pathway inhibitors through nuclear factor κB (NF-κB) dependent expression of the microphthalmia transcription factor (MITF) [40].

Besides cytokines and survival factors secreted by TAMs, extracellular matrix deposition of TAMs also promotes cancer cells resistance to chemotherapy and radiotherapy by remodeling or directing interactions between cancer cells and macrophages [41].

## TAMs promote solid tumor angiogenesis

In solid tumor, vasculature provides oxygenation and nutrition to promote the proliferation of cancer cells. Angiogenesis, known as vascularization, is associated with tumor growth and metastasis, and plays an important role in cancer progression. It has been shown that TAMs are one of the major contributors during the process of forming new vasculature in solid tumor. According to the results from quantitative analysis and evaluation of spatial correlation between TAMs and neovascularization in cervical cancer, TAMs are shown to significantly induce tumor angiogenesis [42]. Macrophage polarization to M2 type is induced by CSF1. Several studies showed that TAMs depletion by inhibiting CSF1 displays substantial attenuation in angiogenic potential and tumor burden in breast cancer [43]. In contrast, when CSF1 level is rescued, TAM depletion is blocked and angiogenic potential is enhanced [4]. All these results suggest that these TAMs are required for tumor angiogenesis. Moreover, several studies have demonstrated that the number of TAMs in colon cancer is positively associated with the number of blood vessels [44]. Besides, the number of infiltrated TAMs and vascular density are showed to be associated with lymph node metastases and prognosis.

Studies have demonstrated that TAMs contribute tumor neovascularization by upregulating VEGF levels [45, 46]. In a skin cancer mouse model, TAM-secreted VEGF-A is reported to induce neoangiogenesis [41]. Indeed, macrophage-produced WNT7b is attributed to increase VEGF-A mRNA and protein expression in vascular endothelial cells, resulting in the angiogenic switch [47]. Another report found that TAMs promote lymphoma vascularization by expressing high levels of VEGF-C in Merkel cell carcinoma, a highly malignant skin neuroendocrine cancer [48]. Hypoxia has been shown to be a key regulator of angiogenesis in solid tumor. Pro-angiogenic functions of TAMs are facilitated by the hypoxia-dependent transcription factor HIF1α (hypoxia-inducible factor 1-α), which transcriptionally upregulate VEGF expression [49]. Additionally, hypoxia can also promote TAMs to infiltrate in the inner region of the tumor by secreting chemokines such as chemokine C-C motif ligand 2 (CCL2), CCL5 and CSF-1.

Neovascularization is also induced by proangiogenic growth factors PDGF and TGF-β secreted by TAMs. Moreover, TAMs increase the production of angiogenesis-related growth factors by inducing pro-inflammatory mediators such as IL-1 and IL-6 [44]. Notably, Weichand et al. indicated that TAMs infiltrate into tumors to promote pulmonary metastasis and tumor lymph angiogenesis by S1PR1 /NLRP3/IL-1β signal in mouse breast cancer model [50]. Apart from PDGF, adrenomedullin and metalloproteinases (MMPs) induced by TAMs are also shown to be involved in angiogenesis. For instance, TAM-derived MMP-9 induces angiogenesis and tumor growth in melanoma [13]. TAMs can also express angiopoietin receptor, endothelial-specific receptor tyrosine kinase TIE2 [51]. Conditional TIE2 gene knockdown in TAMs is sufficient to inhibit tumor angiogenesis and growth in a variety of models [52]. TAMs migrate towards angiopoietin-2, a TIE2 ligand expressed by angiogenic vessels and activated endothelial cells [53]. TIE2 and angiopoietin 2 (ANG2) expression is upregulated by hypoxia, and triggers angiogenesis by establishing an autocrine loop in vascular endothelial cells [52]. TIE2 agonist ANG1 by systemic administration reverses the inhibitory effect of tumor growth caused by REGN910, an angiopoietin-2specific antibody [54].

## TAMs are associated with tumor migration, invasion and metastasis

Tumor cells often leave the primary tumor to create a metastatic colony [55]. The distant metastasis potential of tumor cells depends on the TME. TAMs, the major component of the TME, play crucial roles in cancer metastasis. TAMs primarily facilitate tumor cell invasion and migration by secreting matrix metalloproteinases, serine proteases, and cathepsins which modify cell-cell junctions and disrupt basal membrane [56]. For instance, cathepsin protease activity is induced by IL-4 in TAMs and promotes cancer growth and invasion. In addition, Olga et al. reported that TAM-derived cathepsin B promotes breast cancer cell invasion and lung metastasis [57]. In pancreatic islet cancer, high cathepsin protease activity in TAMs is positively associated with cancer metastases during malignant progression. In pancreatic cancer, TAM-secreted cathepsins B and S induce tumor cell invasion. Recently, Baghel et al. showed that TAM-derived macrophage inflammatory protein-1-β (MIP-1β) induces MYO3A expression and promotes cancer cell matrix protrusive and invasion in breast cancer [58]. In non-small cell lung cancer, Zhang et al. found that TAMs promote cancer cell invasion through TGF-β/SOX9 pathway [59]. Moreover, Yang et al. reported that the infiltration densities of TAMs are significantly higher in breast cancer specimens than in adjacent normal tissue [60]. Moreover, TAMs can promote breast cancer cell migration by secreting CCL18 to upregulate PITPNM3 of cancer cells [61]. In triple-negative breast cancer mouse model, local and systemic levels of MMP-9, VEGF, chitinase-3-like protein 1 (CHI3L1) and Lipocalin-2 (LCN2) induced by TAMs mediate cancer metastasis [62]. It has been reported that TAMs isolated from 98 primary lung cancer tissues express high levels of HGF, cyclooxygenase-2 (COX-2), Cathepsin K, PDGF-B, MMP-9, urokinase-type plasmin activator (uPA) and VEGF-A [63]. Conditioned medium from TAMs significantly promotes cell migration and invasion in various types of human tumor cell lines, while blocking uPA and MMP-9 can inhibit TAM-induced invasion. TAMs enhance tumor cell migration and invasion through a paracrine loop which consists of macrophage-derived epidermal growth factor and tumor-induced growth factor CSF-1 [64]. Consequently, ablation of TAMs by genetic depletion of CSF-1 significantly reduces the number of circulating tumor cells and diminishes metastasis [65].

TAMs also produce several other molecules that promote tumor cell invasion. TAMs upregulate S100A8 and S100A9 expression and promote tumor invasion and migration in colon and Lewis lung carcinoma cells [66]. Secreted protein acidic and rich in cysteine (SPARC) induced by TAMs as a matricellular protein increases tumor extracellular matrix deposition and interaction and thus promotes tumor cell migration [20, 67].

The epithelial-mesenchymal transition (EMT) which is often activated during tumor invasion and metastasis is an important tumor malignant developmental program [68]. TAMs have also been shown to play pivotal roles in cancer EMT. During tumor EMT process, epithelial markers including E-cadherin are decreased in cancer cells, whereas mesenchymal markers such as Vimentin, Beta-catenin, Fibronectin, ZEB1, ZEB2, Slug and Snail are upregulated. Recent studies showed that TAMs decrease E-cadherin, whereas increase Vimentin expression by activating the TLR4/IL-10 signaling pathway in pancreatic cancer cells. It suggests that TAMs promote EMT in pancreatic cancer [69, 70]. In colorectal cancer, the number of infiltrating TAMs is positively associated with Snail expression of cancer cells [71]. Moreover, another study showed that TAMs-derived TGF-β induces colorectal cancer cell EMT via Smad/Snail signaling pathway [72]. Similarly, TAMs promote cancer cells EMT in hepatocellular carcinoma via secreting TGF-β [17]. In breast cancer, TAMs-expressed CCL18 forming a positive feedback loop induces cancer cell EMT [73].

## TAMs in immunosuppression

The regular treatment for solid tumor contains aggressive surgery, radiochemical and hormonal therapy. However, these regular treatments are not tumor-specific but have strong side effects. Cancer immunotherapy that focuses on strengthening the patient’s own immune system to recognize and eliminate tumor cells is currently being applied in clinic. Macrophages are one of the most abundant immune cell populations in the tumor microenvironment. Considerable evidence indicates that macrophages are polarized to a protumoral M2 phenotype [74, 75], which secretes an array of chemokines, cytokines, and enzymes to exert immunosuppression function and downregulate the activation of multiple immune cells. Therefore, TAMs can inhibit immunotherapy effects in solid tumor. Moreover, varieties of chemokines (e.g. CCL2, CCL5, CCL17, CCL18, CCL20 and CCL22), cytokines (e.g. HGF, PDGF-B, VEGF, IL-4, IL-10, prostaglandin [PG] and TGF-β) and enzymes (e.g. Cathepsin K, cyclooxygenase-2 [COX-2], arginase 1 [ARG1] and matrix metalloproteinase [MMPs]) secreted by TAMs can inhibit CD8+ and CD4+ T cells effector function directly. Moreover, these chemokines, cytokines and enzymes derived from TAMs can also stimulate the generation of the induced regulatory T cells (iTregs) and recruit natural Tregs (nTregs), which display immunosuppressive function by directly inhibiting effector T cells or secreting immunosuppressive factors. The differential contributions of iTreg and nTreg to the immunosuppressive properties of TAMs depend on the microenvironment of different tumor types. For example, Curiel *at al.* demonstrated that CCL22 secreted by TAMs recruits CCR4+ nTregs to promote the formation of immunosuppressive microenvironment inhuman ovarian cancer [76]. In colorectal cancer, CCL20 secreted by TAMs recruit CCR6+ nTreg cells [77]. Additionally, immunosuppressive cytokines IL-10 and TGF-β produced by TAMs, induce generation of iTreg by upregulating the pivotal regulatory transcription factor forkhead box P3 (Foxp3) in CD4+ T cells. For example, Denning et al. reported that IL-10 and TGF-β derived from TAM in the intestinal immune system induce iTreg [78]. In turn, Treg cells also promote an M2-like TAM phenotype indirectly and sustain their survival by suppressing CD8+ T cells in tumor microenvironment [79]. For example, nTregs repress CD8+ T cells to decrease production of IFN-γ which promote development and function of TAMs by engaging in fatty acid synthesis of TAMs [79].

The important role of CCL2 in TAM accumulation is supported by the evidences that the levels of tumor-derived CCL2 is correlated with the number of TAMs in several types of tumor, including pancreatic, breast and ovarian cancer [74, 75]. Interestingly, CCL2 secretion has also been detected in TAMs, and contributes to Th2 polarized immunity [80]. In addition, the expression of CCL5 on TAMs is followed by the therapy of tumor. By secreting CCL17, CCL18 and CCL22, TAMs recruit naïve and Th2 lymphocytes and induce ineffective immune responses [81]. Liu et al. demonstrated that conditional macrophage ablation reduces CCL20 levels, blocks CCR6+ nTreg recruitment and suppresses tumor growth in CD11b-DTR mice [77]. In human ovarian carcinoma, CCL22 produced by TAMs mediates trafficking of CCR4+ nTreg cells to the tumor and foster immune privilege [76].

TAMs have also been found to significantly overexpress immunosuppressive cytokines IL-4, IL-10 and TGF-β in human and mouse cancers [82]. IL-10 and TGF-β can also directly modulate T cell functions (Fig. 3). IL-10 suppresses Th1 and Th2 cell functions, whereas TGF-β suppresses the function of cytotoxic T lymphocyte (CTL), Th1 and Th2 cells [82]. L-arginine which is needed for the activation of T cells, was metabolized by ARG1 to urea and L-ornithine. Therefore, TAMs play inhibitory roles on the activation of T cell responses by expressing ARG1 to exhaust L-arginine (Fig. 3). In fact, ARG1 is considered to be an anti-inflammatory M2 macrophage phenotype, and shows a high expression on TAMs [83]. Rodriguez et al. reported that mature tumor-associated myeloid cells (TAMCs) have a high ARG1 expression, and L-arginine depletion in TAMCs inhibits the re-expression of the CD3ζ and antigen-specific proliferation of T cells [84]. Moreover, amino acid metabolism in TAMs causes metabolic starvation of T cells through production of immunosuppressive metabolites by the indoleamine-pyrrole 2,3-dioxygenase 1/2 (IDO1/2) pathway (Fig. 3) [84]. Additionally, hypoxia powerfully augmented the levels of hypoxia-inducible factor (HIF) 1α and 2α in macrophage. HIF1α and HIF2α mediated the immunosuppressive properties of TAMs by upregulating ARG1 and iNOS levels to exhaust arginine and produce NO in TME [85].

In addition to these inhibitory molecules, macrophages express classical and nonclassical MHC class I molecules, cytotoxic T-lymphocyte antigen 4 (CTLA-4) ligand (B7–1 [CD80] and B7–1 [CD86]) and programmed cell death protein 1 (PD-1) ligand 1 (PD-L1) [85]. In general, the function of MHC molecules is presenting antigens to T cells. However, macrophages express the membrane bound or soluble forms of human leucocyte antigen (HLA) molecules (HLA-C, HLA-E and HLA-G) which can suppress the activation of NK cells and T cells upon the molecules bound to the receptor NKG2 [86]. Additionally, HLA-G-transfected antigen-presenting cells inhibit the proliferation of CD4+ T cells, induce their anergy, and cause their differentiation into suppressive cells [87]. Activation of PD-L1 and CD80/86 by their receptors directly inhibits B-cell receptor and T-cell receptor signaling. It has been shown that TAMs in glioblastoma patients had significantly higher expression of PD-L1 compared with healthy donors. Glioma-conditioned media can significantly increase PD-L1 expression in normal monocytes [87]. Analogously, monocytes from patients with hepatocellular carcinoma strongly express PD-L1 and the expression levels of PD-L1 and HLA-DR on tumor infiltrating monocytes have a significant correlation [88]. Moreover, PD-L1+ monocytes inhibit tumor-specific T cell responses. The expression of CD80 and CD86 are expressed on proinflammatory macrophages and are downregulated on anti-inflammatory macrophages [89]. CD80 and CD86 are also the ligands of CD28 on T cell; however, they have a higher affinity with the inhibitory receptor CTLA-4. Additionally, TAMs isolated from human renal cell carcinoma tumors are capable of inducing the expression of CTLA-4 and Foxp3 in T lymphocytes [90]. Further investigation is needed to explore how macrophages on tumor microenvironment are switched from a proinflammatory to an anti-inflammatory.

## Perspectives on TAM-targeted therapeutics

**Fig. 4:**
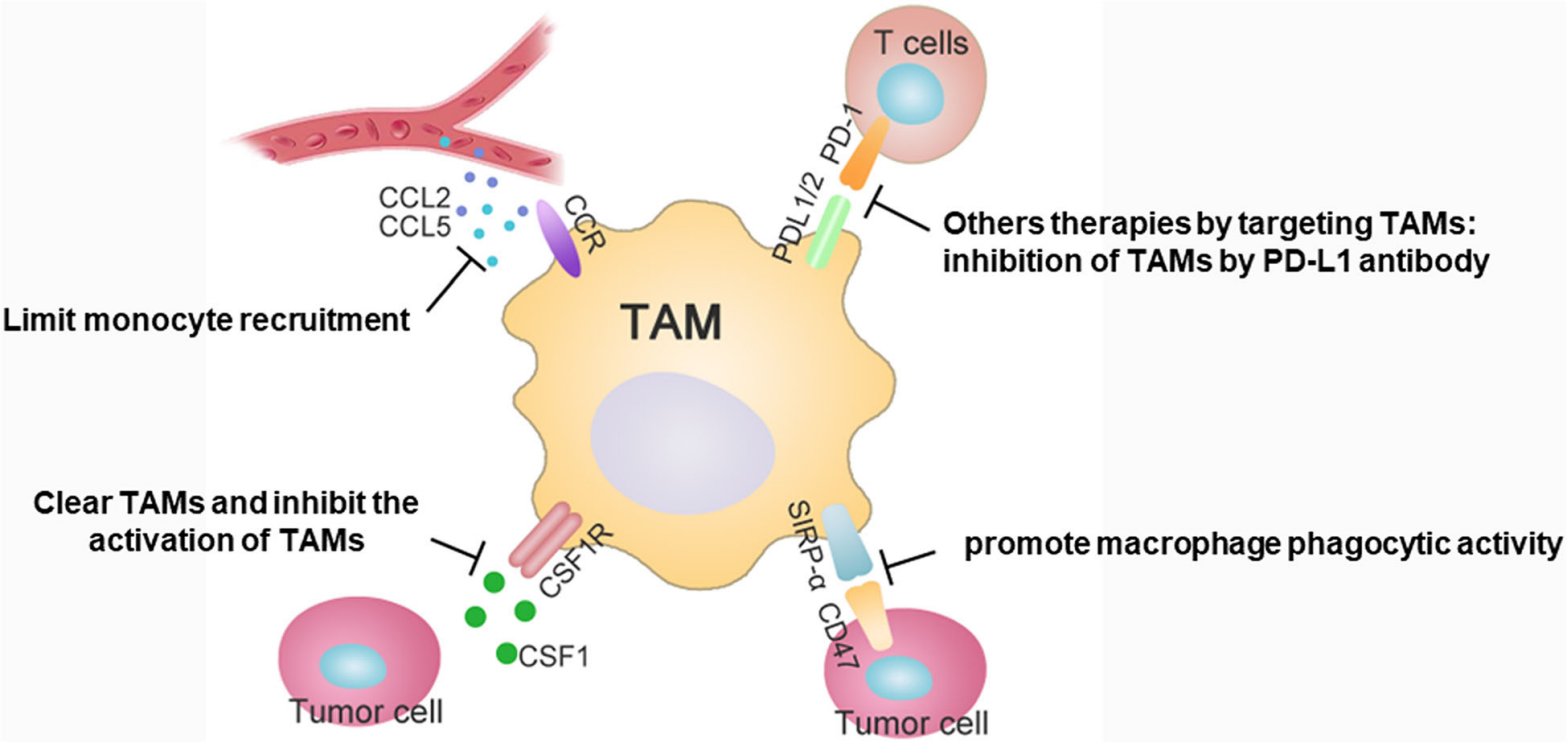
Main therapeutic strategies to target tumor-associated macrophages. The therapeutic strategies include following strategies: 1) clearing macrophages and inhibiting the activation of TAMs like targeting CSF-1/CSF-1R signaling to suppress tumor growth, 2) promoting macrophage phagocytic activity by blocking CD47-SIRP-α signaling, 3) limiting monocyte recruitment via targeting CCL2R, 4) others therapies by targeting TAMs such as inhibition of PD-L1 antibody to promote macrophage phagocytic activity

In many solid tumor types, TAMs are important components of the TME and TAM infiltration is strongly associated with poor clinical outcome of patients. Based on these findings, targeting TAMs is an attractive strategy for solid tumor therapeutic intervention. The therapeutic strategies include clearing macrophages and inhibiting the activation of TAMs, promoting macrophage phagocytic activity, limiting monocyte recruitment and other therapies by targeting TAMs (Fig. 4).

### Clearingmacrophages and inhibit the activation of TAMs

A common strategy of depletion of TAMs is to use liposomal clodronate. Studies showed that liposomal clodronate significantly improves survival in some pre-clinical tumor models [91]. Recently, Piaggio et al. developed novel clodronate-containing liposomes (Clo-Lipo-DOTAP). They showed Clo-Lipo-DOTAP significantly reduces the volume of primary tumors by clearing macrophages in B16/F10 subcutaneous melanoma-bearing mice. In addition, CSF1, a critical macrophage growth factor, plays an important role in monocyte generation and TAMs activation. Therefore, targeting CSF1/CSF1R is an attractive treatment for inhibition of TAMs to suppress tumor development [92]. For example, CSF1 enhances the progression of hepatocellular carcinoma by inducing AIF1 expression in TAMs [93]. Zhu et al. reported that inhibition of CSF1/CSF1R reprograms TAMs and promote the effect of T-cell checkpoint immunotherapy in pancreatic cancer [94]. In murine models, Strachan et al. found that CSF1R blockade delays cervical and breast cancer growth by decreasing the turnover of TAMs and enhancing CD8+ T cells infiltration [43]. However, not all cancer treatments benefit from the depletion of TAMs, especially in the immunotherapy involved in stimulating antitumor innate immunity.

#### To promote macrophage phagocytic activity

In tumor tissue, TAMs are composed of several distinct populations that share features of both M1 and M2 macrophages; however, most studies have shown that TAMs are anti-inflammatory M2 type and correlate with a poor prognosis. Convert of M2 TAMs into M1 proinflammatory macrophages is a potential novel antitumor immunotherapy which is involved in upregulating macrophage phagocytic activity. Two main treatments of modulation of macrophage phagocytic activity are to facilitate antibody-dependent cellular phagocytosis and to inhibit CD47-SIRPα signaling.

Activation of antibody-dependent cellular phagocytosis depends on the interaction between the Fc domain of the antibody and the respective Fc receptor on the membrane of cancer cells. Some monoclonal antibodies approved for the treatment of tumors in clinic have been demonstrated to exert their therapeutic effects, primarily through improving the activity of macrophage phagocytosis. For example, in non-hodgkin lymphoma, rituximab can inhibit tumor development by promoting macrophages phagocytosis [95]. In addition, trastuzumab, a monoclonal antibody drug used for the therapy of HER2-overexpressing breast cancer, has been found to trigger macrophage-mediated phagocytic killing of cancer cells in vitro and in vivo [96].

CD47 block phagocytosis by interacting with SIRPα protein on macrophages to transmit the “don’t eat me” signal. Moreover, CD47 is highly expressed on the cancer cell surface in many tumor types. Therefore, blocking CD47-SIRPα signaling has been found to increase macrophage ability to phagocytose tumor cells. Currently, many therapeutic antibodies and proteins against CD47 and SIRPα have been developed, such as CD47 antibody Hu5F9-G4 and CC-90002.The therapeutic SIRPα protein against CD47 included engineered high affinity SIRPα protein ALX148 and SIRPα-Fc fusion protein TTI-621. Weiskopf et al. reported that CD47 is overexpressed in human small cell lung cancer (SCLC) [97]. Moreover, they showed CD47 antibody Hu5F9-G4 as an immunotherapeutic drug for SCLC can eradicate tumor cells by promoting macrophages phagocytosis. Recently, Petrova et al. showed TTI-621 (SIRPαFc) enhances phagocytosis of both hematologic and solid tumor cells by blocking the CD47-SIRPα axis [97, 98]. Notably, CD47 limits antibody dependent phagocytosis. Therefore, CD47 antibody can enhance the efficiency of phagocytosis induced by monoclonal antibody drug. For example, combination treatment with rituximab and CD47 antibody led to synergetic elimination of lymphoma in mice model [97, 98].

Additionally, some non-antibody drugs also reprogram M2 TAMs to M1 type antitumor macrophages. For example, Yang et al. showed that Pseudomonas aeruginosa mannose sensitive hemagglutinin re-educates M2 TAMs to M1 macrophages to treat malignant pleural effusion treatment in lung cancer patients. Pro-inflammatory M1 macrophage polarization can be induced by iron oxide nanoparticles in tumor tissues [99]. Hydroxychloroquine induces the transition of M2-TAMs to M1-like macrophages, and thus enhance chemo-sensitization and exert lung cancer suppression [100].

#### To limit monocyte recruitment

Since TAMs derive from circulating monocytic precursors, inhibiting monocyte recruitment into tumor tissues is one strategy for targeting TAMs. CCL2 plays an important role in the recruitment and positioning of monocyte in tumors. Targeting the CCL2 and CCL2 receptor (CCR2) is promising treatment for limiting monocyte infiltration and following TAMs generation. In a mouse pancreatic cancer model, CCR2+ monocytes from bone marrow are blocked to mobilize into tumor by PF-04136309, a CCR2 antagonist [101]. Inhibition of monocytes recruitment by PF-04136309 further limits production of TAMs and lead to the inhibition of tumor growth and metastasis. In a phase Ib trial, PF-04136309 has been used to combined with FOLFIRINOX, a combination of the chemotherapy drugs 5-FU, leucovorin, irinotecan and oxaliplatin [102]. In addition, carlumab (CNTO88), an anti-CCL2 monoclonal antibody, has been shown to prevent the development of several cancers in mouse models [103]. Moreover, carlumab combined with chemotherapy showed well tolerance in the treatment of patients with solid tumors. However, carlumab only exert short-term suppression of serum CCL2. Therefore, no significant tumor responses are found [103].

Gone et al. showed that neutralizing CD11b monoclonal antibodies attenuates squamous cell carcinoma growth by preventing the recruitment of myeloid cells into tumors [104]. Moreover, CD11b antibodies trigger a significant enhancement of antitumor response to radiation [105].

#### Other therapies by targeting TAMs

TAMs-targeted therapy in combination with other therapies is more effective relative to single TAMs-targeted therapy. Autophagy, a natural regulated and preserved cellular self-protective mechanism, is characterized by the elimination of the unnecessary or dysfunctional cytoplasmic components by a double-membraned vesicle [106]. Recently, shan et al. showed that autophagy suppresses isoprenaline-induced M2 macrophage polarization via the ROS/ERK and mTOR signaling pathway [107]. Their results suggested target autophagy may play a role in determining the outcomes of tumor treatment by regulating M2 macrophage polarization. PD-L1 and PD-1 expression on TAMs can induce T cell exhaustion and potentially limit the efficacy of T cells associated immunotherapies in solid tumor [108]. Therefore, inhibition of TAMs by PD-L1 antibody likely contributes to T cells-mediated immunotherapies.

## Conclusions

In this article, we specifically reviewed the role of TAMs in solid tumor tumorigenesis, angiogenesis, chemotherapy, radiotherapy, migration, invasion, metastasis and immunosuppression. The well-established mechanisms apart from TAMs-derived factors including cytokines, chemokines and proteases, are also involved in the contact between TAMs and cancer cells. However, the mechanisms need to be studied in greater detail. To target TAMs is a very promising immunotherapeutic strategy. However, the clinical application of current treatment strategy is still very limited. Therefore, it needs to provide more efficacious novel drugs and treatments for future solid tumor therapy.

## Data Availability

Not applicable.

## Abbreviations

ANG: Angiopoietin
ARG1: Arginase 1
CCL: Chemokine (C-C motif) ligand
CHI3L1: Chitinase-3-like protein 1
COX-2: Cyclooxygenase-2
CSF-1: Colony stimulating factor 1
CTLA-4: Cytotoxic T-lymphocyte antigen 4
CXCL: Chemokine (C-X-C motif) ligand
DTR: Diphtheria toxin receptor
EMT: Epithelial-mesenchymal transition
ERα: Estrogen receptor α
Foxp3: Forkhead box P3
HGF: Hepatocyte growth factor
HIF1α: Hypoxia-inducible factor 1-α
HLA: Human leucocyte antigen
IDO1/2: Indoleamine-pyrrole 2,3-dioxygenase 1/2
IFN: Interferon
IL: Interleukin
iTregs: Induced regulatory T cells
LCN2: Lipocalin-2
MAPK: Mitogen-activated protein kinase
M-CSF: Macrophage colony-stimulating factor
MDSC: Myeloid-derived suppressor cells
MFG-E8: Milk-fat globule-epidermal growth factor-VIII
MITF: Microphthalmia transcription factor
MIP-1β: Macrophage inflammatory protein-1-β
MMPs: Metalloproteinases
MMTV: Mouse mammary tumor virus
NKG2: Natural killer cells receptor
nTregs: Nature regulatory T cells
PD-1: Programmed cell death protein 1
PDGF: Platelet-derived growth factor
PD-L1: Programmed cell death protein 1 ligand 1
PG: Prostaglandin
PITPNM3: Phosphatidylinositol transfer protein, membrane-associate family member 3
PR: Progesterone receptor
PyMT: Polyoma middle T
15-PGDH: 15-hydroxyprostaglandin dehydrogenase
RAB22A: Ras-related protein Rab-22A
ROS/RNS: Reactive oxygen species/reactive nitrogen species
SPARC: Secreted protein acidic and rich in cysteine
TGF-β: Transforming growth factor β
TLRs: Toll-like receptors
TNF-α: Tumor necrosis factor-α
TSC: Tumor stem cells
VEGF: Vascular endothelial growth factor
